# Urinary extracellular vesicular release of aquaporins in patients with renal transplantation

**DOI:** 10.1186/s12882-019-1398-7

**Published:** 2019-06-11

**Authors:** Sayaka Oshikawa-Hori, Naoko Yokota-Ikeda, Hiroko Sonoda, Masahiro Ikeda

**Affiliations:** 1 Department of Veterinary Pharmacology, Faculty of Agriculture, University of Miyazaki, Gakuenkibanadai-Nishi 1-1, Miyazaki, 889-2192 Japan; 2 Nephrology, Miyazaki Prefectural Miyazaki Hospital, Kitatakamatsu 5-30, Miyazaki, 880-8510 Japan

**Keywords:** Extracellular vesicles, Renal transplantation, Aquaporin-1, Aquaporin-2, Kidney, Urine, Exosomes

## Abstract

**Background:**

Diuresis has been observed within a week following renal transplantation, suggesting that the procedure causes acute disturbance of renal water homeostasis. Aquaporin (AQP) 1 and AQP2, important proteins for renal water reabsorption, have been identified in urinary extracellular vesicles (uEV-AQP1 and -AQP2), and experimental studies have shown that the presence of uEV-AQP1 and -AQP2 may be an indicator of their levels of expression in the kidney. However, the release patterns of uEV-AQP1 and -AQP2 during the acute phase following renal transplantation are largely unknown.

**Methods:**

In this study, we examined the release of uEV-AQP1 and -AQP2 in recipients until 6 days (day 6) after renal transplantation. At Miyazaki prefectural Miyazaki Hospital, Japan, uEVs were obtained from 7 recipients, all of whom had received renal allografts from living donors. uEVs were isolated by differential centrifugation.

**Results:**

Immunoblotting analysis showed that the release of uEV-AQP2 was significantly decreased on day 1 in comparison with a control sample (from 3 healthy volunteers), accompanied by high urine output and low urine osmolality. Thereafter, the level increased gradually to the control level by day 6. The release pattern of uEV-AQP1 was similar to that of uEV-AQP2, but the levels did not reach statistical significance in comparison with the control level at any of the time points examined. Evaluation of the relationship between urinary osmolality and uEV-AQPs revealed a significant correlation for uEV-AQP2, but not for uEV-AQP1.

**Conclusion:**

These results indicate that acute diuresis after renal transplantation might be due to a decrease in the renal expression of AQP2, whose level can be estimated from the amount released in uEVs.

## Background

Following the introduction of renal transplantation in the 1950s, diuresis in the recipients had been occasionally observed within a week after the operation [1, 2]. However, the underlying mechanisms had remained largely unknown because the molecular mechanism responsible for urinary concentration had yet to be clarified. In 1992, Agre’s group discovered aquaporin-1 (AQP1), which is now known to play critical roles in water reabsorption in the renal proximal tubule and in maintaining the renal medullary osmotic gradient [3, 4]. One year later, Sasaki’s group found another AQP, AQP2, which is regulated by vasopressin, and subsequent studies have revealed that this protein is involved in the mechanism of urinary concentration in collecting ducts [4, 5]. Currently, thirteen AQPs (AQP0–12) are known to exist in mammals, of which eight (AQP1–4, 6–8, 11) are expressed in renal epithelial cells [4].

Extracellular vesicles (EVs) are composed of exosomes, microvesicles, and apoptotic bodies, among which exosomes and microvesicles have been a focus of intense translational research with the aim of developing novel biomarkers and therapeutics for renal disease [6–10]. Exosomes, small EVs (30–150 nm in diameter), are derived from internal vesicles enclosed in multivesicular bodies (MVBs). When the MVBs fuse with the plasma membrane, internal vesicles are released as exosomes into extracellular fluids such as blood and urine. Microvesicles are larger EVs with a diameter of 50–2000 nm, budding directly from the plasma membrane. Because of the structural similarity of exosomes and microvesicles, and the lack of appropriate markers that can discriminate between them, the International Society for Extracellular Vesicles has recommended the use of the generic term “EVs” for all released vesicles [8, 10], and we adopted this recommendation in the present study.

EVs are known to selectively contain functional proteins from their cells of origin, and their release has also been observed to depend on the state of their original cells [11–15]. Among the various AQPs, AQP1 and AQP2 have been identified in urinary EVs (uEVs), and several studies with animal models have shown that their levels in uEVs are related to their levels in the kidney [15–17].

Several experimental studies have shown that AQP1 and AQP2 might be involved in the diuresis evident during the acute phase after renal transplantation [18–20]. On the other hand, information from humans is still very limited. We have observed that the release of AQP1-bearing uEVs (uEV-AQP1) was decreased in a renal transplant recipient 48 h after surgery [16]. However, to our knowledge, no other information is available, and the release patterns of uEV-AQP1 and -AQP2 in the acute phase of renal transplantation in humans remain largely unknown.

In order to characterize the release patterns of uEV-AQP1 and -AQP2 after renal transplantation, we examined seven recipients in the acute phase after surgery. We also measured marker proteins for uEVs including tumor susceptibility gene 101 (TSG101) protein and apoptosis-linked gene 2-interacting protein X (Alix) [15, 21].

In the present paper, since uEVs are produced through the above-mentioned mechanisms, we use the term “release” for uEVs such as hormones and autacoids.

## Methods

### Study participants

This study was approved by the Miyazaki Prefectural Miyazaki Hospital Institutional Review Board in accordance with the Ethical Guidelines for Clinical Studies in Japan (Miyazaki of Health, Labour and Welfare, July 30, 2003, Amended December 28, 2004). Spot urine samples were obtained from 7 patients (2 males aged 42 and 52 and 5 females aged 25, 43, 53, 59, and 62) on days 1, 2, and 6 after receiving renal allografts from living donors (including one case of pre-emptive renal transplantation) at Miyazaki Prefectural Miyazaki Hospital Institution between 2009 and 2011. All of the patients were treated with a triple immunosuppressive regimen including tacrolimus (0.15–0.3 mg/kg/day, Astellas Pharma Ltd., Tokyo, Japan), mycophenolate mofetil (2 g/body/day, Novartis, Basel, Switzerland), and methylprednisolone (8 mg/body/day, Sanofi Aventis, Paris, France). Also, basiliximab (20 mg/body/day, Novartis) was added on days 0 and 4.

We employed a standard form of fluid management, including infusion during the operation and compensatory infusion in accordance with urine output after the operation. Since the patients had rather limited water intake during the first 24 h after the operation, compensatory infusion was carefully performed. Thereafter, their water intake increased. When the patients were able to drink well and their water balance was maintained, the infusion was withdrawn.

A spot urine sample from donor was also collected on days 1, 2, and 6 in some cases. Control spot urine samples were also collected from 3 healthy male volunteers aged 21 to 46 years. A sample made from the three healthy volunteers was always used to normalize quantification in each gel. Urine osmolality was measured using an automatic osmometer (Osmostation om-6060, Arkray, Kyoto, Japan).

### Isolation of uEVs

Isolation of uEVs was performed as described previously [16]. Briefly, just after collection, each urine sample was mixed with a protease inhibitor mixture (final concentration, 1 mM EDTA, 0.5 mM p-amidinophenyl methanesulfonyl fluoride hydrochloride, and 0.12 mM leupeptin). Thereafter, the urine was centrifuged at 1000 x g at 4 °C for 15 min to eliminate urinary debris. The supernatant was centrifuged at 17,000 x g at 4 °C for 30 min. The resulting supernatant was then ultracentrifuged at 200,000 x g (Optima TL Ultracentrifuge, Beckman Instruments, CA) at 4 °C for 1 h to isolate a low-density membrane fraction. Many studies use the term exosomes to refer to the EVs in this low-density membrane fraction [10]. After the ultracentrifugation, a protease inhibitor mixture was added to the pellet. Subsequently, the suspended pellet was solubilized in 4 × sample buffer (8% SDS, 50% glycerol, 250 mM Tris-HCL, 0.05% bromophenol blue, 400 mM DTT, pH 6.8), then incubated at 37 °C for 30 min and stored at − 80 °C until use.

### Gel electrophoresis and immunoblot analysis

Immunoblot analysis was performed as described previously [16]. Briefly, each urine sample was loaded with an equal amount of creatinine per lane, and separated by SDS-PAGE. For the detection of each protein, we used the following primary and secondary antibodies: anti-AQP1 (catalog no. sc-20810, Santa Cruz Biotechnology, Santa Cruz, Dallas, TX), anti-AQP2 (catalog no. sc-9882, Santa Cruz Biotechnology), anti-TSG101 (catalog no. ab83–100, Abcam, Cambridge, UK), anti-Alix (catalog no. sc-49268, Santa Cruz Biotechnology), anti-rabbit IgG (catalog no. 7074, Cell Signaling Technology, Danvers, MA), anti-mouse IgG (catalog no. 1858413, Thermo Fisher Scientific, Waltham, MA), and anti-goat IgG (catalog no. P0449, Dako Japan, Tokyo, Japan). Antibody-antigen interactions were visualized using the SuperSignal West-Femto Chemiluminescence detection system (Thermo Fisher Scientific, Waltham, MA). Quantitative analysis of the resulting bands was performed using the WinRoof software package version 5.7 (Mitani, Tokyo, Japan).

### Statistical analysis

Box plots were generated using the BoxPlotR: a web tool for generation of box plots (http://boxplot.tyerslab.com) [22]. Differences between renal transplant patients and controls were analyzed by the Mann–Whitney *U* test or, when the sample mean should be compared with a hypothesized population mean, one sample t-test using EZR (Saitama Medical Center, Jichi Medical University, version 1.29) (http://www.jichi.ac.jp/saitama-sct/SaitamaHP.files/statmedEN.html) on R commander (version 2.1–7), which is a graphical user interface for R (The R Foundation for Statistical Computing, version 3.2.1) [23]. Statistical analysis of correlations between osmolality and uEV-AQPs was performed using Pearson’s correlation test. All values were considered to be statistically significant at *P* < 0.05.

## Results

**Fig. 1:**
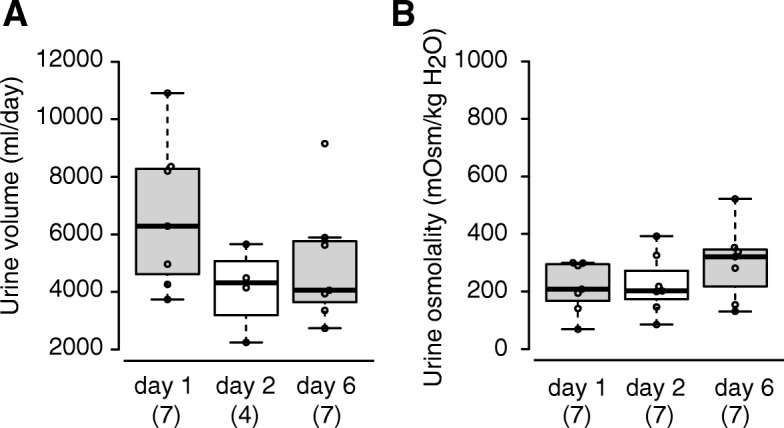
Urinary volume and osmolality in renal transplant recipients. Dot and box plots of urine volume (**a**) and osmolality (**b**) are shown for recipients on days 1, 2, and 6. The central line, top border and bottom border represent the median, 25th and 75th percentiles, and the whiskers show 1.5 times the interquartile range from the lower and upper percentiles. Only four samples were available for measurement of urine volume on day 2. Parentheses indicate the numbers of patients

Blood parameters in renal transplant recipients at 1 (day 1), 2, and 6 days after surgery are shown in Table 1. The levels of blood urea nitrogen (BUN) in these patients were within the normal range (8.6–22.9 mg/dl) at all of the time points examined. The serum creatinine (SCr) concentration on day 1 was higher and then decreased to the normal range (male, 0.6–1.2 mg/dl, female, 0.4–1.0 mg/dl). Figure 1 summarizes data for daily urine volume and urine osmolality. Urine output on day 1 was markedly higher than normal (500–2000 ml/day), and thereafter decreased but still remained high. Similarly, urine osmolality was obviously low in comparison with the normal range (580–1130 mOsm/kg H2O) at any of the time points examined, especially on days 1 and 2. In fact, when we compared the urinary osmolality of the patients with our pooled data from healthy volunteers (477–946 mOsm/kg H2O, *n* = 9), urine osmolality in the recipients was significantly lower at any of the time points examined (unfortunately, urine volume data for healthy volunteers were not available). On the other hand, on days 1 and 2, the water balance was maintained by isotonic infusion (see Methods). These data suggested that the patients we had recruited exhibited acute diuresis after renal transplantation, being most prominent on day 1.

**Table 1:** BUN and SCr concentrations in recipients

	Day 1	Day 2	Day 6
BUN (mg/dl)	18.6 ± 4.0 (7)	12.2 ± 2.6	15.1 ± 3.2
SCr (mg/dl)	2.6 ± 0.7 (7)	1.2 ± 0.2	1.0 ± 0.2

Values are represented as means ± SE. Parentheses indicate the numbers of patients

**Fig. 2:**
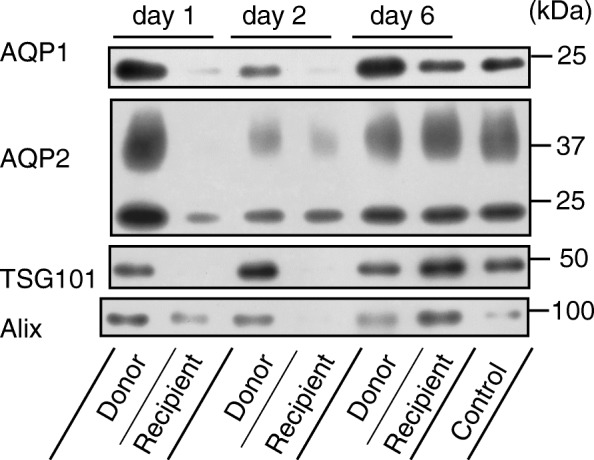
Representative immunoblot images of AQP1, AQP2, TSG101, and Alix in urinary extracellular vesicles. Representative immunoblot images of AQP1 (uEV-AQP1), AQP2 (uEV-AQP2), TSG101 (uEV-TSG101), and Alix (uEV-Alix) in urinary extracellular vesicles in a donor and recipient. Urine was collected from each individual on days 1, 2, and 6. The control sample was made from 3 healthy volunteers

**Fig. 3:**
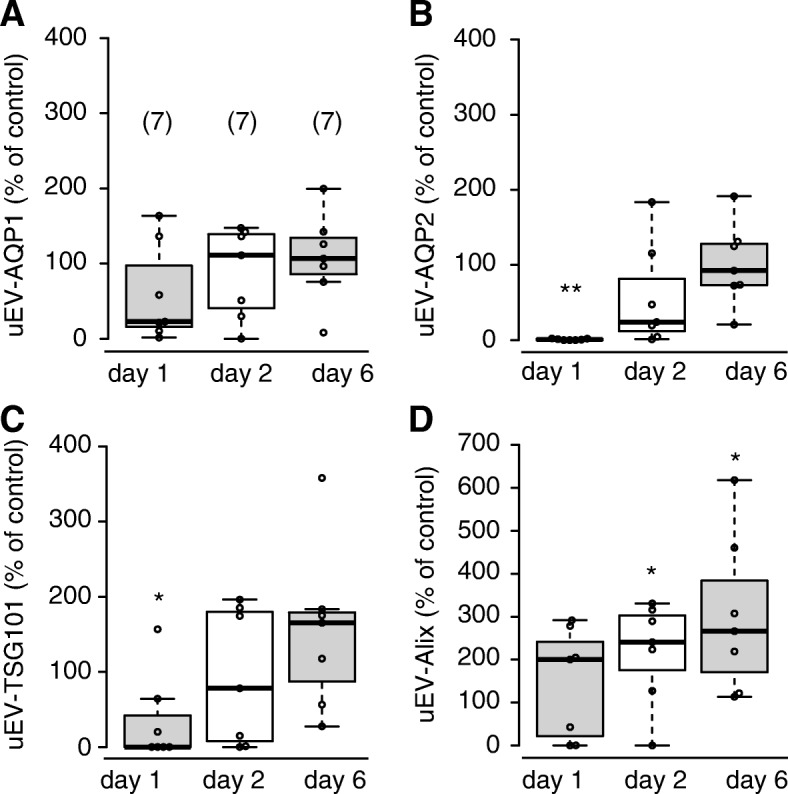
Dot and box plots of uEV-AQP1, uEV-AQP2, uEV-TSG101, and uEV-Alix in recipients. Dot and box plots of release of uEV-AQP1 (**a**), uEV-AQP2 (**b**), uEV-TSG101 (**c**), and uEV-Alix (**d**) on days 1, 2, and 6 in recipients. Each value is calculated as a percentage of the level of the control sample. **P < 0.01 and *P < 0.05, compared with the value of 100% (one sample t- test)

Figure 2 shows a representative immunoblot of uEV-proteins in a recipient and a donor. The levels of AQP1 and AQP2 in the recipient were reduced in comparison with those of the control (shown on the far right) on days 1 and 2, while on day 6 the levels recovered to the control level. The level of TSG101 showed alterations similar to those of AQPs, being somewhat higher than the control level on day 6. The level of Alix was decreased on day 2 and increased on day 6. In the donor, the levels of AQP1 and AQP2 were moderately decreased on day 2. Figure 3 summarizes the data from the immunoblot analyses of the recipients. Release of uEV-AQP2 in recipients was significantly decreased on day 1. On day 2, in 5 out of 7 patients, a decreased release of uEV-AQP2 was observed, but the decrease did not reach significance. The level on day 6 was comparable to the control level. For AQP1, a similar tendency was observed but did not reach statistical significance in comparison with the control level at any of the examined time points. Release of uEV-TSG101 was significantly decreased on day 1 and the level on day 6 tended to be increased. For Alix, the levels were increased at all of the time points examined, becoming statistically significant on days 2 and 6 in comparison with the control level.

**Fig. 4:**
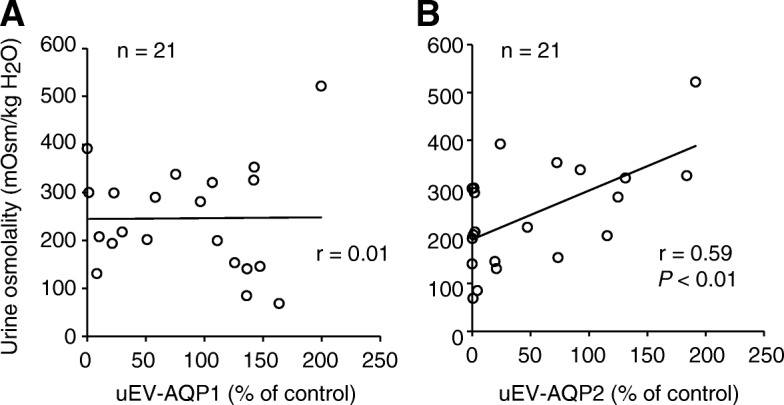
Correlation between urinary osmolality and release of uEV-AQP1 or uEV-AQP2. The relationships between urinary osmolality and release of uEV-AQP1 (**a**) or -AQP2 (**b**) for all samples (on days 1, 2, and 6) are shown. The line is the least-squares regression line. n and r indicate the number of individuals and the correlation coefficient, respectively. There is a significant correlation between urinary osmolality and the release of uEV-AQP2, but not that of uEV-AQP1 (Pearson analysis, *P* < 0.01)

Next, we examined the correlation between urine osmolality and release of uEV-AQP1 or -AQP2 using all of the data from recipients (Fig. 4). This revealed a significant relationship between osmolality and release of uEV-AQP2, but not that of uEV-AQP1.

## Discussion

One major finding in this study was that release of uEV-AQP2 might be applicable as a biomarker of the urinary concentration defect in renal transplant recipients. A significant reduction in the release of uEV-AQP2 was observed on day 1, accompanied by high urinary output and low urine osmolality. Thereafter, the release of uEV-AQP2 gradually increased to the control level, along with recovery of the urinary concentration mechanism. Furthermore, a significant correlation between urinary osmolality and release of uEV-AQP2 was observed. On the other hand, the correlation for AQP1 was not significant. These data indicate that release of uEV-AQP2 might be an indicator of the urinary concentration defect in recipients within a week after renal transplantation.

Previously, we observed a decrease in release of uEV-AQP2 in rats treated with gentamicin [17]. Along with this decrease, we also detected a defect in the urinary concentration mechanism and polyuria, and a reduced level of renal AQP2 expression. Although the present study did not examine the renal expression level of AQP2, our previous observation strongly suggests that the decrease in the release of uEV-AQP2 in recipients was due to the reduced renal expression of AQP2.

In the present study, tacrolimus was used to suppress the immunoreaction after renal transplantation. Tacrolimus is known to inhibit phosphatase 2B [24], which would be expected to enhance the phosphorylation of AQP2. Increase in the phosphorylation of AQP2 in the renal collecting duct cells would stimulate the translocation of AQP2 from intracellular vesicles to the apical membrane, and the expression of AQP2 protein through increased transcription [4]. Therefore, treatment of patients with tacrolimus is considered to affect the release of uEV-AQP2. However, several studies have shown that tacrolimus had little effect on both the phosphorylation and the expression of AQP2 in the renal collecting duct cells [24, 25], and thus the contribution of tacrolimus to the release of uEV-AQP2 was considered to be minimal.

In contrast to the release of uEV-AQP2, that of uEV-AQP1 tended to be decreased on day 1, but the decrease did not become statistical significant in comparison with the control group. Several experimental studies have revealed that warm renal ischemia-reperfusion dramatically decreases the renal expression of AQP1 [26, 27], suggesting a decrease in the release of uEV-AQP1 after renal ischemia-reperfusion. On the other hand, in the human renal transplantation setting, renal ischemia-reflow has been performed at a low temperature in comparison with the above experimental models. It has been reported that a lower temperature reduces the severity of renal ischemia-reperfusion injury [28]. Therefore, a possible reason for the non-significant release of uEV-AQP1 on day 1 may have been the different temperature conditions for renal ischemia-reperfusion between humans and experimental animals.

In the present study, the release patterns of uEV-TSG101 and -Alix, both of which have been used as exosomal marker proteins [15, 21], did not show a comparable tendency. The release of uEV-TSG101 was significantly reduced on day 1 and then increased to above the control level. On the other hand, the release of uEV-Alix was higher at all of the time points examined in this study relative to the control group. Although the reason for this discrepancy is currently unclear, one possibility may be that factors other than the number of exosomes released into urine affected the release of either uEV-TSG101 or -Alix. Since Alix is involved in cell death signaling as an apoptosis inducer [29], such signaling might be activated at all time points. In order to develop proteins in uEVs as a diagnosis tool, marker proteins in the uEVs would be important as an internal control for estimating the number of exosomes, and therefore further studies will be needed to clarify the internal control proteins that might be appropriate.

Our present findings suggest that uEV-AQP2 might be a useful marker for estimation of renal AQP2 dysregulation, thus affecting urinary concentration ability in recipients after renal transplantation. Recently, the beneficial effect of water intake has been discussed in terms of preservation of kidney function in patients with certain types of kidney disease through suppression of the plasma level of vasopressin [30]. Since AQP2 is a vasopressin-regulated water channel protein [4, 5], uEV-AQP2 as a marker of renal water handling function might be useful for determining whether water intake therapy might be appropriate. Although direct measurement of blood vasopressin is problematic, as most of it (more than 90%) binds to platelets and is unstable in collected plasma, it would be important in a future study to evaluate the relationship between uEV-AQP2 and plasma copeptin, which has been shown to be a useful surrogate marker of vasopressin [31].

## Conclusion

This study has clearly provided information on the relationship between release of uEV-AQP2 and the renal urinary concentration defect in the early phase after renal transplantation. However, this results were limited by the small sample size. Further studies with larger samples may provide more accurate information concerning the usefulness of this marker in renal transplantation.

## Data Availability

The datasets for this study are available from M.I. on reasonable request.

## Abbreviations

Alix: Apoptosis-linked gene 2-interacting protein X
AQP: Aquaporin
MVBs: Multivesicular bodies
TSG101: Tumor susceptibility gene 101
uEV: Urinary extracellular vesicle
